# A novel signature based on cancer-associated fibroblast genes to predict prognosis, immune feature, and therapeutic response in breast cancer

**DOI:** 10.18632/aging.204685

**Published:** 2023-05-04

**Authors:** Yichen Wang, Wenchang Lv, Yi Yi, Qi Zhang, Jun Zhang, Yiping Wu

**Affiliations:** 1 Department of Plastic Surgery, Tongji Hospital, Tongji Medical College, Huazhong University of Science and Technology, Wuhan 430030, Hubei, China; 2 Department of Thyroid and Breast Surgery, Shenzhen Qianhai Shekou Free Trade Zone Hospital, Shenzhen 518067, Guangdong, China

**Keywords:** breast cancer, cancer-associated fibroblasts, prognosis signature, therapeutic targets, immune infiltration

## Abstract

Breast cancer (BC) ranks first in the incidence of tumors in women and remains the most prevalent malignancy in women worldwide. Cancer-associated fibroblasts (CAFs) in the tumor microenvironment (TME) profoundly influence the progression, recurrence, and therapeutic resistance in BC. Here, we intended to establish a risk signature based on screened CAF-associated genes in BC (BCCGs) for patient stratification. Initially, BCCGs were screened by a combination of several CAF gene sets. The identified BCGGs were found to differ significantly in the overall survival (OS) of BC patients. Accordingly, we constructed a prognostic prediction signature of 5 BCCGs, which were independent prognostic factors associated with BC based on univariate and multivariate Cox regression. The risk model divided patients into low- and high-risk groups, accompanied by different OS, clinical features, and immune infiltration characteristics. Receiver operating characteristic (ROC) curves and a nomogram further validated the predictive performance of the prognostic model. Notably, 21 anticancer agents targeting these BCCGs possessed better sensitivity in BC patients. Meanwhile, the elevated expression of the majority of immune checkpoint genes suggested that the high-risk group may benefit more from immune checkpoint inhibitors (ICIs) therapy. Taken together, our well-established model is a robust instrument to precisely and comprehensively predict the prognosis, immune features, and drug sensitivity in BC patients, for combating BC.

## INTRODUCTION

Breast cancer (BC) is the most prevalent malignancy among women worldwide [1]. In recent years, despite the vigorous development of comprehensive treatment strategies, including surgery, chemotherapy, hormonal therapy, and immunotherapy, the issues of recurrence, metastasis, chemotherapy drug resistance, and other poor prognosis remain non-negligible challenges in the management of BC patients [2]. The highly complex and heterogeneous nature of BC limits the wide applicability of existing BC staging. Therefore, the exploration of novel biomarkers is imperative to provide new possibilities for risk prognosis and individualized treatment of BC patients.

Tumor malignant progression is thought to be irrefutably related to alterations in the tumor microenvironment (TME) [3]. Activated fibroblasts, vascular endothelial cells, pericytes, adipocytes, immune cells, and abundant extracellular stromal cells together constitute the complex TME [4]. Cancer-associated fibroblasts (CAFs) are one of the most abundant stromal components in TME and are major participants in tumor-stromal crosstalk. A large body of evidence implies that CAFs contribute to the promotion of tumor development. In addition to directly promoting the growth and invasion of cancer cells, CAFs can also affect angiogenesis, connective tissue formation, hypoxia, immunomodulation, and epithelial mesenchymal transition (EMT), thus driving tumor metastasis and drug resistance [5].

Consequently, identifying the matrix components in TME not only enables precise targeting of CAFs, but also makes it a potentially promising target for BC therapy. Multiple studies have described the prognostic significance of CAF-associated biomarkers in BC. For example, BC patients with a higher proportion of Alpha-smooth muscle actin (α-SMA) positive fibroblasts exhibited shorter overall and relapse-free survival [6]. α-SMA was also identified as a novel biomarker of trastuzumab resistance in HER2-positive BC [7]. Similarly, the CAF marker, platelet-derived growth factor beta (PDGFRβ) receptor, was associated with considerably shorter recurrence-free survival and brain metastases in BC [8, 9]. Conversely, fibroblast activating protein (FAP) is another classical CAF marker, exhibiting a significant relationship on longer overall and disease-free survival for BC [10]. However, several analyses of the prognostic value of the CAF marker podoplanin have yielded conflicting conclusions [11–13]. Additionally, different studies on caveolin-1 (Cav1) had also come to contradictory conclusions [14–16]. Hence, the specific relationship between the reactive stroma indicated by various CAF markers and prognostic factors has not been fully elucidated.

**Figure 1:**
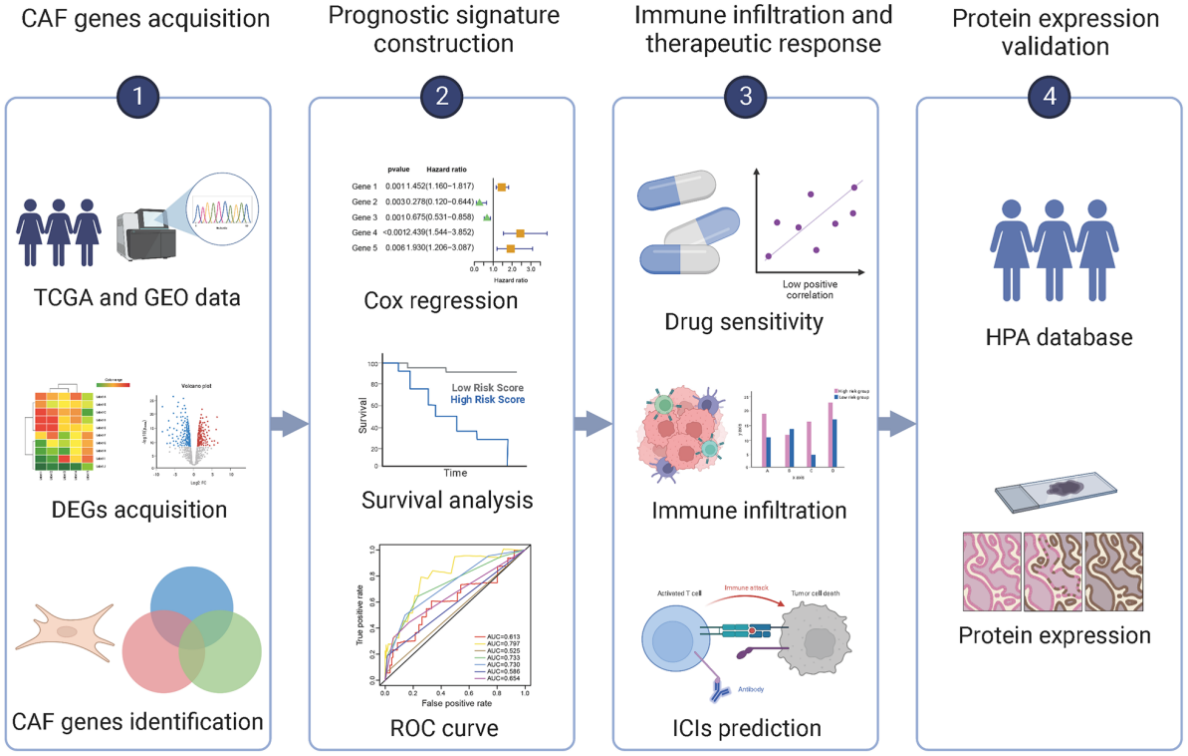
Flow chart of this study.

CAFs are substantially a group of cell subpopulations with spatial, phenotypical, and functional heterogeneity [17]. This complexity suggests that a comprehensive CAF gene profile is appropriate for characterizing CAFs, subsequently defining the prognosis and optimizing clinical diagnosis and treatment of BC patients. Therefore, the characterization of CAFs by gene combinations is a promising direction for combating BC. Accordingly, here, we constructed and validated a risk prognosis signature based on CAF-associated genes in BC (BCCGs) by comprehensive bioinformatics analysis. A collection of BCCGs associated with OS survival in BC patients was identified based on Kaplan-Meier (K-M) survival analysis. Then 5 BCCGs were further identified for constructing a prognostic risk score model to predict BC prognosis. The risk score derived from BCCGs could predict the sensitivity of anticancer drugs and immunotherapy response, thus contributing to the precise treatment of BC. In addition, there was a substantial association between the immune infiltration landscape of TME and different risk scores, which might contribute to synergistic effects in CAF-targeted treatment and immunotherapy. These findings provide new insights into the prognostic prediction methods and strategies for future individualized therapeutics of BC patients. The entire flow of this study was shown in Figure 1.

## RESULTS

### Identification of BCCGs

**Figure 2:**
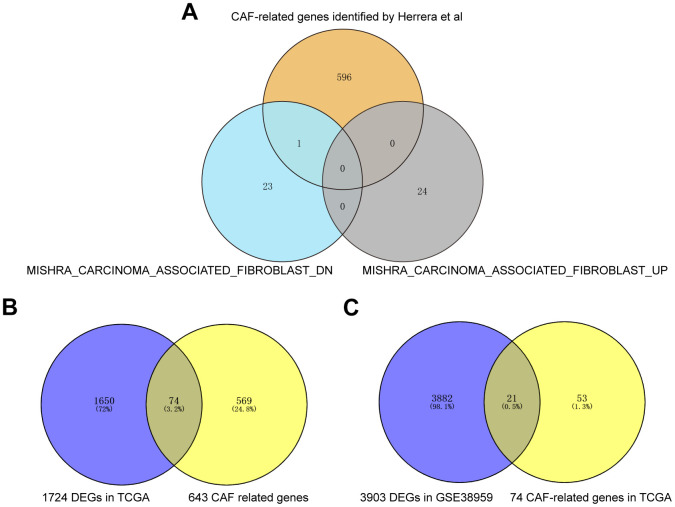
**Acquisition of BCCGs in BC.** (**A**) Three CAF gene sets were collected to obtain a comprehensive pool of CAF-associated genes. (**B**) CAF-related gens overlapped with DEGs in TCGA to obtain BCCGs. (**C**) BCCGs were further validated in GSE38959.

Firstly, 643 CAF-related genes were obtained by taking the full set of 3 CAF-related gene sets to eliminate overlapping genes (Figure 2A). Then, 1,724 differentially expressed genes (DEGs) were mined from TCGA by “DESeq2” R package and “GEO2R”. These 1724 DEGs were crossed with 643 CAF-related genes, and 74 differentially expressed BCCGs were identified (Figure 2B). Then, 3924 DEGs were mined from GSE38959, and the final 21 BCCGs were filtered by the further intersection of 3924 DEGs in GSE38959 with 74 BCCGs obtained from the previous intersection set (Figure 2C).

### Association of 21 BCCGs with the survival of BC patients

**Figure 3:**
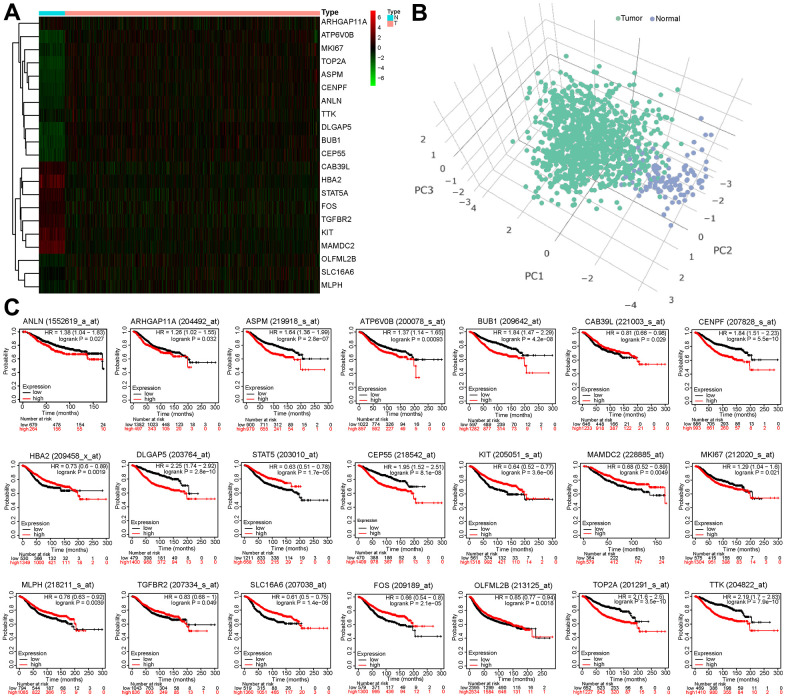
**Survival analysis of 21 BCCGs in BC.** (**A**) Heatmap displayed different expression patterns of the 21 BCCGs in the BC and control groups. (**B**) PCA analysis based on the 21 BCCGs could distinctly divide the BC group and control group. (**C**) K-M survival analysis of the 21 BCCGs in BC patients.

In Figure 3A, the heatmap showed that the expression of the 21 BCCGs was significantly different in normal and tumor samples. PCA analysis based on these 21 BCCGs could distinctly classify the BC patients and normal individuals into two clusters (Figure 3B). Therefore, we further analyzed the relationship between these 21 BCCGs and the prognosis of BC patients (Figure 3C). K-M survival analysis exhibited that all the 21 genes were associated with survival in BC patients. The low expression of ALIN, ARHGAP11A, ASPM, ATP6V0B, BUB1, CENPF, DLGAP5, CEP55, MKI67, TOP2A, TTK was more favorable to the long-term survival of BC patients, while the high expression of CAB39L, HBA2, STAT5, KIT, MAMDC2, MLPH, TGFBR2, SLC16A6, FOS, OLFML2B was more associated with the long-term survival of BC patients. This suggested that it was feasible to use the CAF genes to stratify the prognosis of BC patients.

### Establishment of a prognostic model based on CAF-related risk score

**Figure 4:**
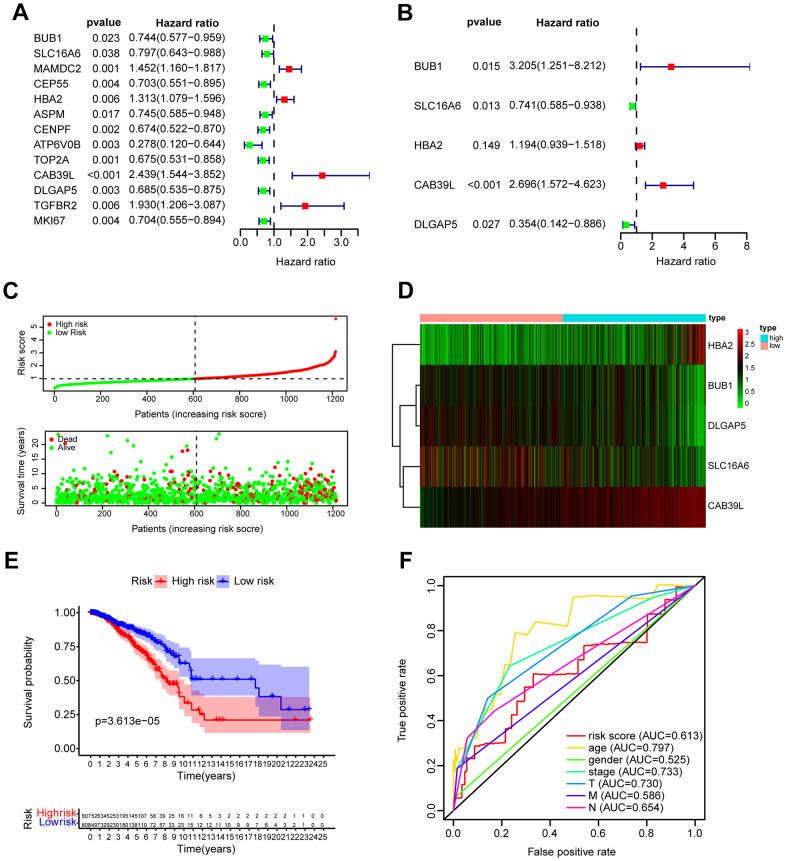
**Construction and validation of prognostic model based on BCCGs.** (**A**) The association between clinical prognosis and potential BCCGs was established by univariate Cox regression. (**B**) Multivariate Cox regression analysis revealed the underlying predictive BCCGs of BC. (**C**) The survival status distribution of BC patients after grouping by median risk score. (**D**) Expression profile of the 5 BCCGs of BC patients. (**E**) K-M curves of patients assigned to low- and high-risk groups. (**F**) ROC curves of risk score and other clinicopathological characteristics.

To establish a CAF-related prognostic model, we first screened 13 genes of prognostic value from 21 BCCGs by univariate Cox regression analysis (Figure 4A), in which BUB1, SLC16A6, CEP55, ASPM, CENPF, ATP6V0B, TOP2A, DLGAP5, MKI67 were favorable factors for patient prognosis (HR < 1), while MAMDC2, HBA2, CAB39L, TGFBR2 were unfavorable factors (HR > 1). Multivariate Cox regression further identified 5 BCCGs based on the minimum AIC value as BUB1, SLC16A6, HBA2, CAB39L, and DLGAP5 to construct the prognostic model (Figure 4B). Each patient obtained the risk score value by the above formula. Then we divided the patients into low- and high-risk groups according to the median risk score, and the survival time of patients became shorter with the increasing risk score (Figure 4C). The expression pattern of the 5 BCCGs was distinctly different between the low-risk group and high-risk group (Figure 4D). Subsequently, K-M analysis showed that the overall survival (OS) of patients in the high-risk group was shorter than those in the low-risk group (Figure 4E), and the results (Figure 4F) demonstrated that the AUC of the ROC curve with risk scores was 0.613.

### Correlation of clinicopathological features with risk scores

**Figure 5:**
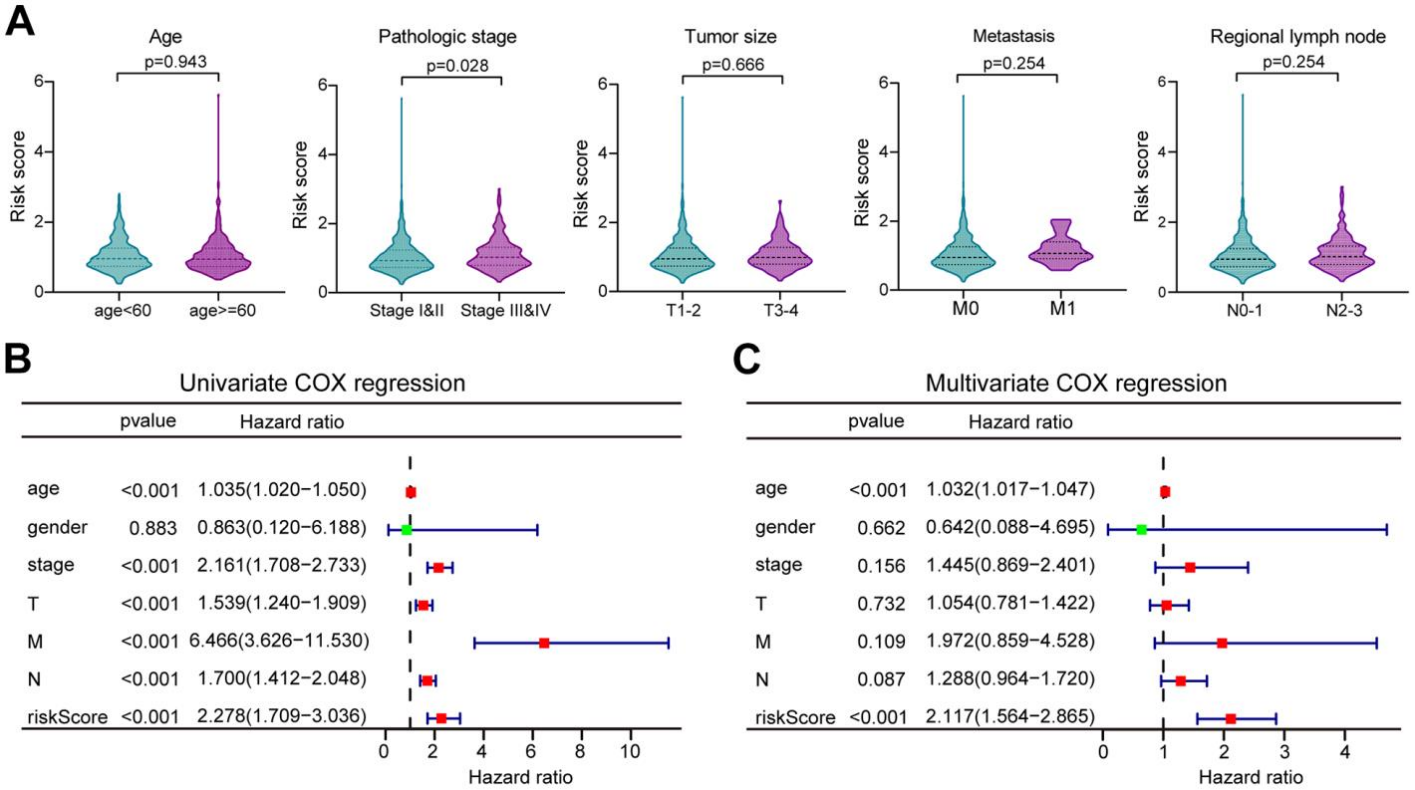
**The correlation of clinicopathological characteristics with risk score.** (**A**) The risk score distribution between BC patients with different clinicopathological features. (**B**) Univariate Cox regression analysis of risk scores and other clinical features in forest plots. (**C**) Multivariate Cox regression analysis of risk scores and other clinical features in forest plots.

The distribution of risk scores in the corresponding samples was investigated according to age, clinical stage, tumor size, regional lymph node, and distant metastasis levels. Higher risk scores were associated with the higher clinical stage (P = 0.028), but not with the age or TNM of the tumor (Figure 5A). Univariate Cox regression analysis was performed for age, gender, clinical stage, tumor size (T), regional lymph nodes (N), distant metastases (M), and prognostic risk score. The findings suggested that age, clinical stage, T, N, and M were all prognostic correlates (P < 0.05, Figure 5B). But further multivariate Cox regression implied that only age and risk score were independent prognostic factors for BC (P < 0.05, Figure 4C).

### Construction of a predictive nomogram for validating the prognostic model

**Figure 6:**
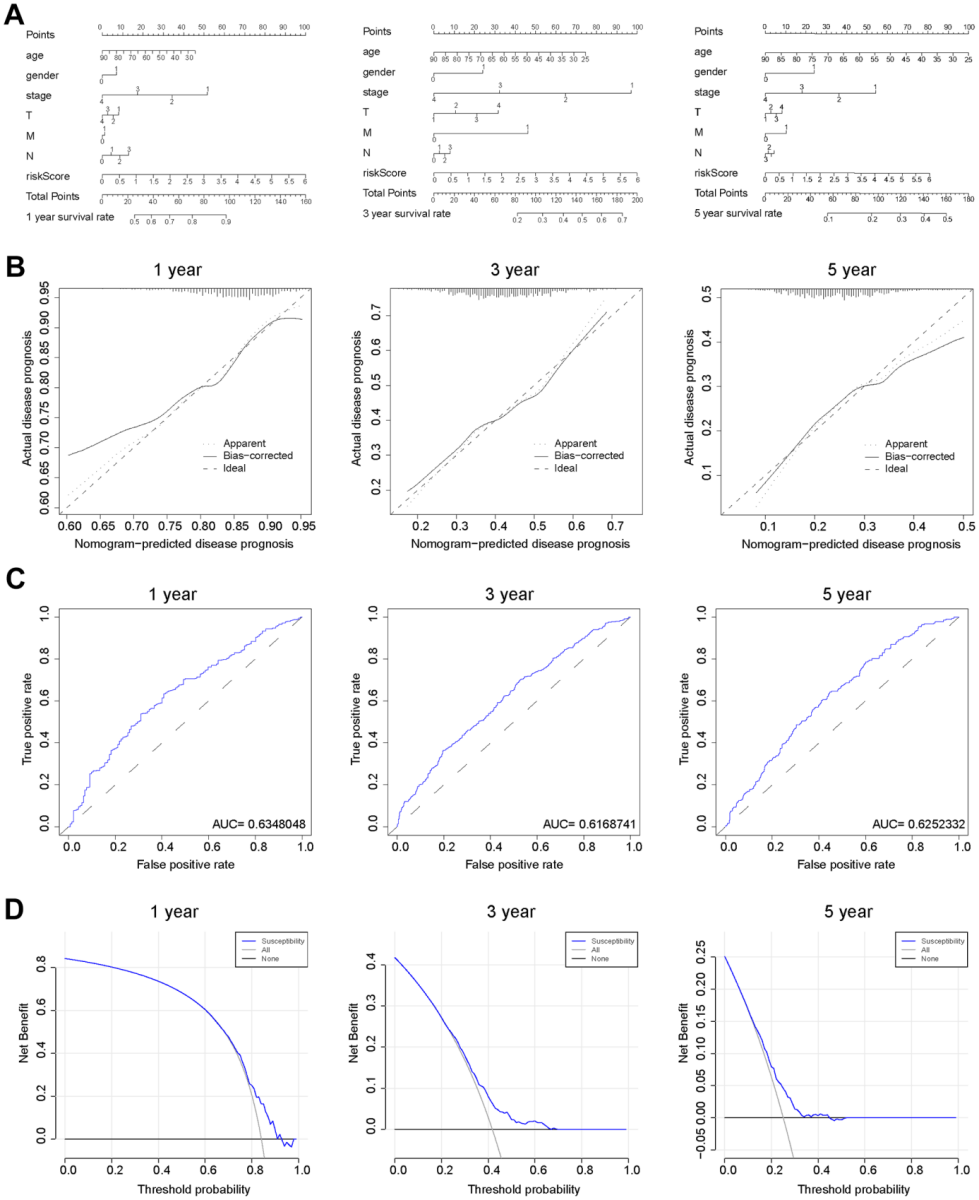
**Verification of the predicting capability of prognostic model based on BCCGs.** (**A**) Nomogram model build on CAF-related risk score and clinicopathological features to predict 1-, 3-, and 5-year OS of BC patients. (**B**) Calibration curves for the nomogram revealed qualify the predictive accuracy and ability. (**C**) ROC curves of the prognostic model of BC patients for 1-, 3- and 5-year. (**D**) The DCA of a prognostic model for 1-, 3- and 5-year overall survival, respectively.

As nomograms are widely utilized to predict patient survival, here we constructed a nomogram that could predict the probability of survival at 1-, 3-, and 5-year (Figure 6A). The length of the risk score line segment reflected the greater contribution of the risk factor to outcome event. The performance of the risk signature could further be quantified in terms of calibration, which revealed relatively good predictive accuracy between actual and predicted probability (Figure 6B). In addition, the 1-, 3-, and 5-year ROC curves showed the corresponding AUC for risk score were 0.635, 0.617, and 0.625, respectively (Figure 6C). Furthermore, decision curve analysis (DCA) calculated a clinical “net benefit” for the prediction models in comparison to default strategies of treating all or no patients at 1-, 3- and 5-year (Figure 6D). Hence, it concluded that, this risk signature was a well-performed predictive parameter. In summary, the prediction potential of prognostic nomogram was verified from multiple perspectives.

### Prediction of anticancer agents sensitivity based on CAF gene expression

**Figure 7:**
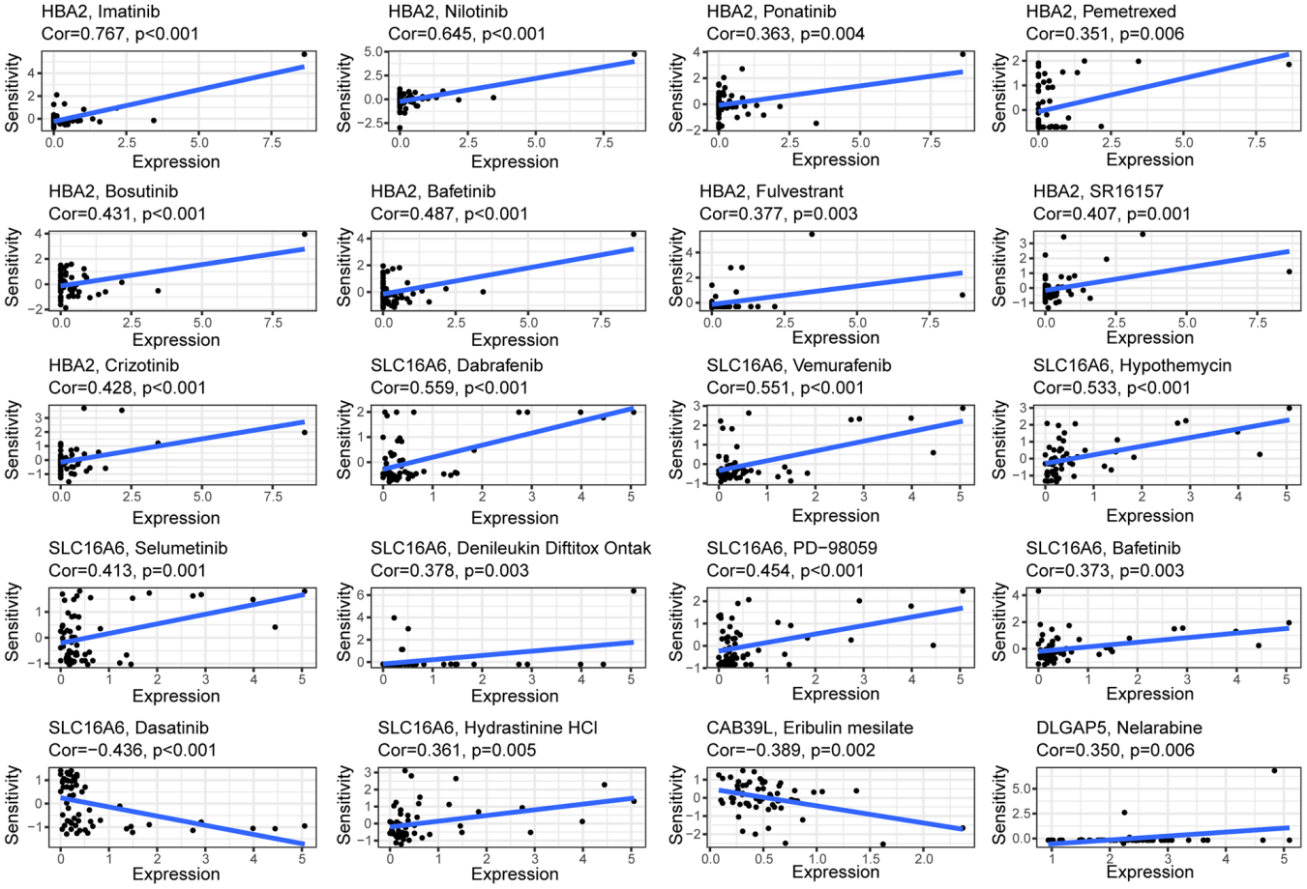
Correlation between the expressions of BCCGs and sensitivity of potential anticancer drugs: The relationship between the IC50 of various medications and the risk score.

The heterogeneity of BC tumors and individual variability of patients often render conventional treatments ineffective in achieving desired outcome in all patients. Therefore, identifying the molecular characteristics of tumors and determining appropriate therapeutic agents is a key challenge for precision medicine. CellMiner is a suite of genomics and pharmacology-based tools for screening effective anti-cancer drugs [18]. Here, we explored the relationship between 5 BCCGs and chemotherapeutic drug sensitivity using the Cellminer database in this study (Figure 7). The expression of HBA2 was found to be positively correlated with the sensitivity of Imatinib, Nilotinib, Ponatinib, Pemetrexed, Bosutinib, Bafetinib, and Fulvestrant. The expression of SLC16A6 demonstrated a positive correlation with the sensitivity of Dabrafenib, Vemurafenib, Hypothemycin, Selumetinin, Denileukin Diftitox Ontak, PD-98059, Bafetinib, and Dasatinib. Besides, the higher the expression of CAB39L, the worse the drug sensitivity of BC patients to Eribulin mesylate, meanwhile the higher the expression of DLGAP5, the better the sensitivity of BC patients to Nelarabine.

### Different immune landscapes in low- and high-risk group

**Figure 8:**
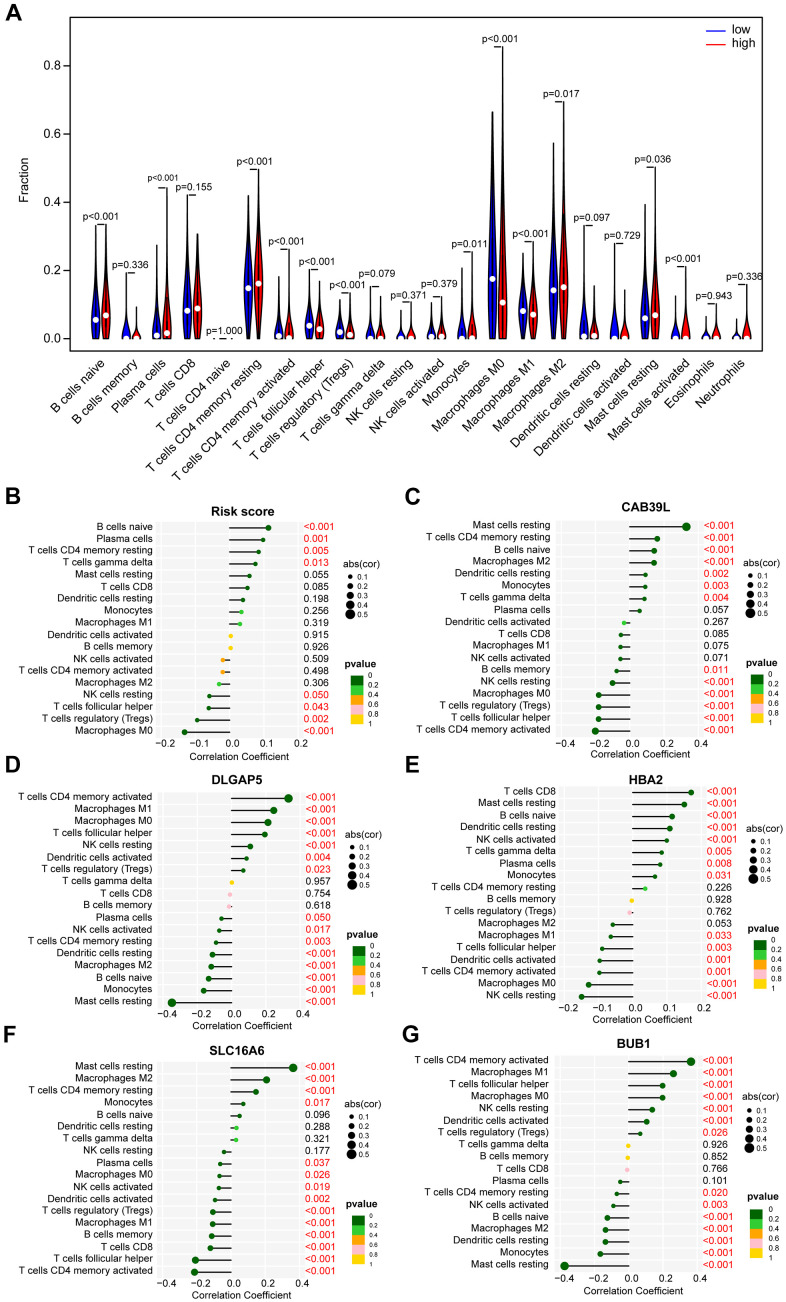
**The distinct immune infiltration features of TME between low- and high-risk groups.** (**A**) Distribution of the relative abundance of immune cells in different risk groups. (**B**) Correlation analysis of immune infiltrating cells and risk scores. (**C**) Correlation analysis of immune infiltrating cells and CAB39L. (**D**) Correlation analysis of immune infiltrating cells and DLGAP5. (**E**) Correlation analysis of immune infiltrating cells and HBA2. (**F**) Correlation analysis of immune infiltrating cells and SLC16A6. (**G**) Correlation analysis of immune infiltrating cells and BUB1.

CAFs are an essential component of TME, and the immune infiltration characteristics of TME are closely linked to tumorigenesis, invasion, and metastasis. Accordingly, the CIBERSORT algorithm emphasized that B cells naïve, Plasma cells, T cells CD4 memory resting, Monocytes, Macrophages M2, Mast cells resting, and Mast cells activated were mainly enriched in the high-risk group, while T cells CD4 memory activated, T cells follicular helper, T cells regulatory (Tregs), Macrophages M0, and Macrophages M1 were more frequently found in the low-risk group (Figure 8A). We further assessed the relationship between immune components and risk scores. There was a significant positive correlation between B-naïvenaive, plasma cells, T-cell CD4 memory, T-cell gamma delta, and risk score, but a negative relationship between NK-cell resting, T-cell follicular helper, T-cell regulatory (Tregs), macrophage M0, and risk score (Figure 8B). In addition, each of the 5 BCCGs constituting the risk score was significantly correlated with the immune cell score (Figure 8C–8G).

### Immunotherapy response prediction

**Figure 9:**
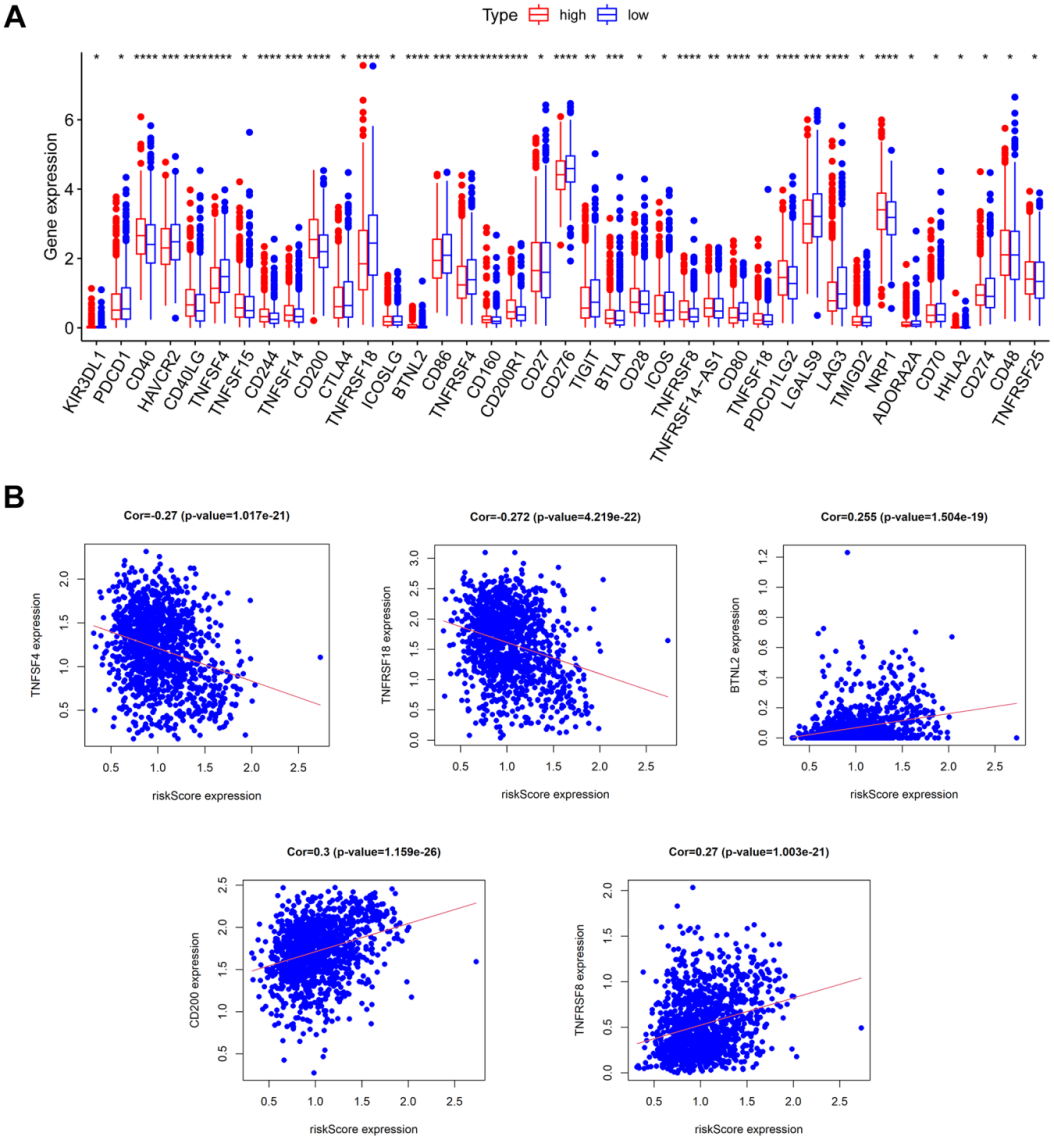
**Prediction of immunotherapy response.** (**A**) The expression of immune checkpoints in low- and high-risk groups. (**B**) The relationship between risk score and the expression of some immune checkpoints.

Immunotherapy is rapidly emerging for its specificity, safety, and effectiveness, and BC is considered one of the tumor types that can benefit from immunotherapy. Immune checkpoint inhibitors (ICIs) are a huge breakthrough in BC treatment. To explore the correlation between CAF-related characteristics and immunotherapy response, we compared the differential expression of immune checkpoints between the low- and high-risk groups. There were 39 out of 43 immune checkpoint genes with significant differences in expression in the low- and high-risk groups (Figure 9A). Most immune checkpoints, including CD40, CD40LG, TNFSF15, CD244, TNFSF14, and CD200, were upregulated in the high-risk group. In addition, we discovered an inverse relationship between the risk score and expression of TNFSF4, TNFRSF18, and BTNL2, and a positive relationship between the risk score with expression of CD200 and TNFRSF8 (Figure 9B). These results suggested that there might be a correlation between risk score and immunotherapy and that the high-risk group was more likely to benefit from ICIs therapy.

### Functional enrichment analysis

**Figure 10:**
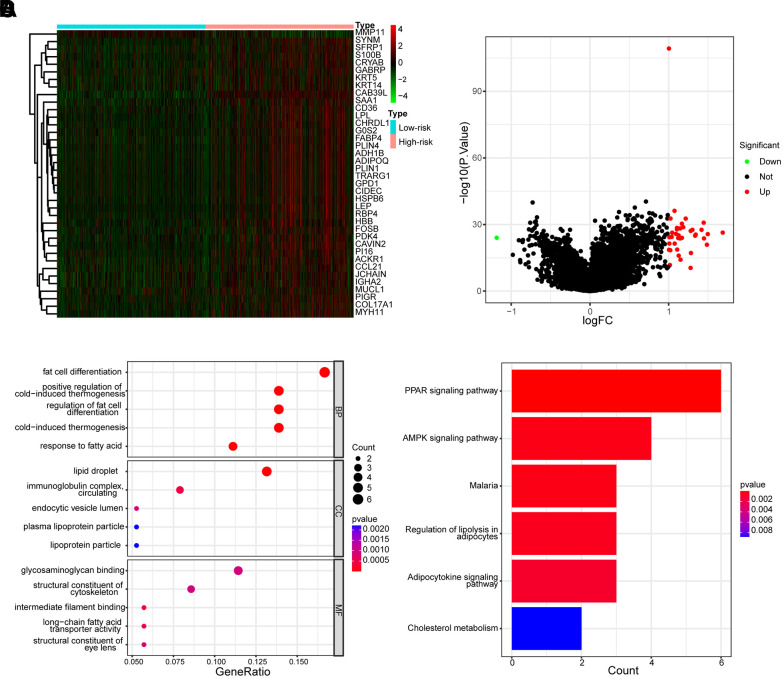
**Different gene expression profiles and functional analysis of low- and high-risk groups.** (**A**) The heatmap of the expression differences in low- and high-risk groups. (**B**) The volcanic map depicted dysregulated genes between low- and high-risk groups. (**C**) The enrichment map illustrated GO annotation analysis of DEGs between low- and high-risk groups. (**D**) The enrichment map illustrated the KEGG pathway analysis of DEGs between low- and high-risk groups.

To further explore the differences in gene expression patterns between the low- and high-risk groups, we analyzed the molecular processes and biological pathways between the two groups. The heatmap (Figure 10A) and volcano map (Figure 10B) exhibited the differential gene expression profiles between the low- and high-risk groups. The Gene Ontology (GO) analysis revealed that the most enriched Biological Processes (BP) were fat cell differentiation, positive regulation of cold-induced thermogenesis, and regulation of fat cell differentiation, the most enriched Cellular Component (CC) were lipid droplet, immunoglobulin complex circulating and endocytic vesicle lumen, the most enriched Molecular Function (MF) were glycosaminoglycan binding, structural constituent of the cytoskeleton and intermedia filament binding (Figure 10C). Furthermore, The Kyoto Encyclopedia of Genes and Genomes (KEGG) enrichment analysis showed that the differential genes in the low- and high-risk groups might be involved in the PPAR signaling pathway, AMPK signaling pathway, Regulation of lipolysis in adipocytes, Adipocytokine signaling pathways and cholesterol metabolism (Figure 10D).

### Expression pattern of CAF gene signature in BC tissue

**Figure 11:**
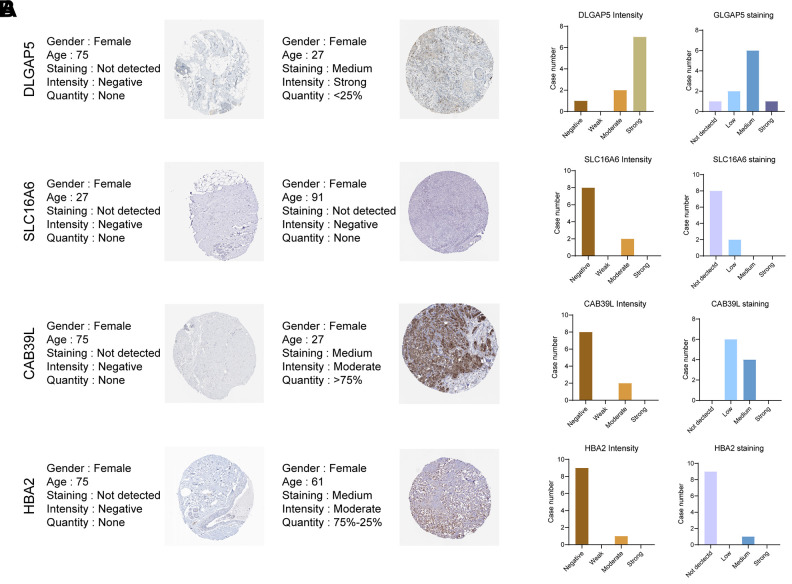
**Validation of the 5 BCCGs in normal breast tissue and BC tissue.** (**A**) IHC staining of protein expression for 5 BCCGs. (**B**) Bar charts represent IHC staining and intensities for five BCCGs.

To further explore the important role of CAF-related gene signatures in BC, we analyzed protein expression patterns of BCCGs in normal and tumor samples in the Human Protein Atlas (HPA) database (Figure 11A). Immunohistochemistry (IHC) results from HPA demonstrated that DLGAP5 protein was found to be strongly expressed in BC tumor tissues, while SLC16A6, CAB39L, and HBA demonstrated lower protein expression in BC tissues (Figure 11B). The differential expression of these BCCGs in the BC group and the control group suggests that they might play an important role in the initiation and progression of BC.

## DISCUSSION

CAFs are a heterogeneous group of cells in terms of cellular origin, phenotype, and function, and are also the most important components of TME. Activated CAFs can facilitate tumor growth, angiogenesis, invasion, and metastasis, as well as extracellular matrix (ECM) remodeling and chemoresistance through multiple mechanisms. Multiple studies have documented the critical role of CAFs in BC development and progression. For instance, Cohen et al. discovered that fibroblasts drove the immunosuppressive and growth-promoting microenvironment of BC through secreting Chitinase 3-like 1 [19]. Besides, fibroblast-derived IL-33 was found to promote BC metastasis by altering the immune microenvironment and driving type 2 immunity [20]. In addition, Wen et al. demonstrated that IL-32 secreted by CAFs promoted BC cell invasion and metastasis through integrin β3-p38 MAPK signaling [21]. Furthermore, CAF-derived inflammasome was reported to promote tumor progression and metastasis by regulating the TME in an immunosuppressive environment [22]. The characteristics and roles of CAFs in BC have been gradually uncovered, and CAFs have been considered as potential targets for anticancer therapy. Therefore, a comprehensive understanding of the molecular characterization and functional properties of CAFs in BC is necessary for targeted therapy of CAFs. However, most studies focused on the role of single CAF-related gene regulators in BC, and the combined effects of CAF-related genes have rarely been elucidated. The characteristics of CAF cell subpopulations dictate that an integrated gene expression pattern might be more appropriate to describe CAFs. The integrated characterization of multiple genes is more conducive to diagnostic optimization, prognostic prediction, and therapeutic guidance for BC patients.

A Comprehensive gene expression pattern is more suitable to describe the CAF population and is subsequently used for disease prediction. A set of stroma-derived prognostic predictors is applied to stratify BC prognosis [23]. Several ECM-related genes have also been found to be associated with survival and risk of recurrence in BC [24]. Oliver Frings et al. described a gene expression signature based on PDGF-activated fibroblasts that could be used to identify BC and showed independent and strong prognostic significance [25]. Although each of the aforementioned genes had considerable prognostic power, the overall effect of multiple CAF-associated gene regulation had not been fully described. Secondly, there is little genetic overlap between these CAF features, so the new molecular tool is expected to help elucidate tumor-mesenchymal interactions for individualized prognostic assessment.

The application of high-throughput technology enables a more comprehensive characterization of tumor stroma and expands the selection range of CAF biomarkers. Here, this study originally investigated the prognostic significance of a CAF-associated gene pattern in BC patients. In this study, we created a BC prognostic risk signature based on 5 CAF-associated genes and confirmed their prognostic value. Firstly, to improve the comprehensiveness and reliability of the results, a combination of the GEO database, TCGA database, and GSEA database was used to perform the screening of the CAF-related gene set. Next, we identified 5 independent BCCGs independently associated with BC prognosis by univariate Cox regression and multivariate Cox regression analysis, namely BUB1, SLC16A6, HBA2, CAB39L, and DLGAP5. Then, we constructed the corresponding risk score prognostic model by 5 BCCGs, which could accurately predict the prognosis of BC patients, as demonstrated by longer OS in the low-risk group than in the high-risk group. In addition, the combination of the prognostic risk score model with prognosis-related clinicopathological features assists to ameliorate the predictive power and clinical applicability of the model. Ultimately, there was indeed a difference in gene function analysis between the low-risk and high-risk groups, which further confirmed that the use of risk stratification to classify the prognosis of BC patients was theoretical.

The signature-constructing BCCGs have been acknowledged more or less in BC progression. BUB1 was found to be variably expressed in BC cell lines as early as 2000 [26]. Later, an analysis of 1858 primary BC patients recognized that BUB1 was a key kinase in low-grade luminal BC, and that low expression of BUB1 accounted for a poor prognosis of BC patients [27]. Recently, metformin treatment reduced oncogenic miR-21-5p in BC cells, further releasing CAB39L expression and evoking activation of AMPK, which was closely associated with reduced migratory and invasive capacity in BC cell lines [28]. Moreover, HBA2 was significantly downregulated in BC tissues and displayed excellent diagnostic performance [29]. DLGAP5 is a microtubule-associated protein and mitotic phosphorylated substrate of Aurora-A. Elevated gene expression of DLGAP5 was reported to be associated with poor OS in BC patients and might be a useful target for BC diagnosis and treatment [30]. Furthermore, the genetic signature constituted by the transporter protein SLC16A6 and other factors is involved in prognostic prediction, immune infiltration, and immunotherapeutic response in BC [31]. Therefore, the risk signatures based on these 5 BCCGs have a theoretical foundation.

A key goal of precision medicine is to match drugs to the genomic determinants of response. Identifying molecular features of tumors that influence response to specific drug therapy is particularly challenging because of patient diversity, tumor heterogeneity, and incomplete knowledge of the multiple molecular determinants of response. Therefore, we leveraged the CellMiner database to predict candidate drugs that were highly associated with the CAF gene. The results of our screening suggested that these different types of chemotherapeutic agents respond differently to different risks. Differences in sensitivity to anticancer agents between low- and high-risk groups further help optimize personalized treatment for BC patients.

CAFs interact with tumor-infiltrating immune cells as well as other immune components, by secreting various cytokines, growth factors, chemokines, and exosomes, consequently resulting in an immunosuppressive TME that allows cancer cells to escape from the surveillance by the immune system [4]. Future targeted immunotherapies may benefit from in-depth research of CAFs and their interactions with the immunological milieu, particularly the complex pathways that link CAFs with immune cells. Although various studies have highlighted the importance of BCCGs, there is a dearth of research involved in the immune TME and immunotherapy of BCCGs. We further evaluated the immune distribution between low- and high-risk groups. There was a significant difference in stromal immune scores between the low- and high-risk groups. Notably, higher levels of activated CD4+ T cell infiltration showed better survival outcomes [32], while T-cell follicular helper-related activity was significantly enhanced in BC clusters with better prognosis [33]. In the present work, CD4+ and T-cell follicular helper infiltration were indeed significantly increased in the low-risk group. In addition, the enrichment of M1 cells helps to protect BC patients [34], while a higher proportion of M2 cells is a risk factor for BC patients [35], which was also consistent with our results. The risk scores including 5 corresponding BCCGs were also significantly correlated with various immune cell fractions. Furthermore, the differential expression of immune checkpoint genes suggested that patients in the high-risk group might be more sensitive to its treatment.

Although we deciphered a comprehensive feature and obtained an excellent predictive capability, there are still some issues that warrant to be addressed at present. Firstly, this study was a retrospective research based on the existing TCGA, GEO, and other databases. In a further prospective study, more information and sample collections obtained from real-world substances are needed to investigate the potency of this signature. Secondly, we have preliminarily indicated the excellent abilities of this signature in judging the BC strategies. Thus, there is a lack of more credible validation within *in vivo* or *in vitro* experiments. Thirdly, more investigations are needed to identify the molecular mechanisms of CAFs during BC progression. In addition, more extensive multicenter clinical trials are important to confirm the efficacy of immunotherapy regimens targeting CAFs.

In conclusion, by manipulating a series of integrated bioinformatic methods, this signature was successfully constructed based on 5 specific screened BCCGs, including BUB1, SLC16A6, DLGAP5, CAB39L, and HBA2. This signature exhibited robust, reliable, and comprehensive capabilities in predicting the prognosis, immune feature, and drug sensitivity for better combating BC. Taken together, our study provides a feasible strategy for the stratification and individualized treatment of BC patients.

## MATERIALS AND METHODS

### Public data collection

RNA sequencing data, including normal breast samples and BC samples, were obtained from The Cancer Genome Atlas (TCGA) database (https://portal.gdc.cancer.gov/) and Gene Expression Omnibus (GEO) database (https://www.ncbi.nlm.nih.gov/geo/). The inclusion criteria for the sample were as follows: (a) diagnosed with histologically confirmed BC; (b) available survival data; (c) complete clinical information. There were 1101 BC samples and 572 control samples in the TCGA database. GSE38959 included 30 BC samples and 13 normal breast samples.

### Acquisition of CAF-associated genes

We used the term “fibroblast” as a keyword for searching in the MSigDB database (https://www.gsea-msigdb.org/gsea/msigdb/index.jsp). Two genomes associated with CAF (MISHRA CARCINOMA ASSOCIATED FIBROBLAST UP and MISHRA CARCINOMA ASSOCIATED FIBROBLAST DN) were eventually obtained for subsequent analysis. Besides, a gene set, including 596 CAF-associated genes determined by Herrera et al. from a previous study in 2021 was also included in the study [36]. The full gene sets of the three were taken, and 643 CAF-associated genes were acquired after deleting duplicates.

### Acquisition of CAF-associated genes in BC (BCCGs)

The DEGs in TCGA were intersected with the 643 CAF-associated genes to obtain 74 BCCGs. Then the 74 BCCGs intersected with GSE38959 DEGs to further narrow down the range of BCCGs. Ultimately, we obtained a gene set of 21 BCCGs.

### Establishment of a prognostic risk model

K-M survival estimate was conducted to assess the relationship between these 21 BCCGs and the survival of BC patients. Then, univariate Cox regression analysis was used to further screen out key prognostic BCCGs. Next, multivariate Cox regression analysis identified BCCGs showing independent prognostic associations, and a risk score model was developed to predict the prognosis of patients with TCGA BC patients. Risk scores were calculated for each sample based on a risk score formula = (1.164664003* expression level of BUB) + (-0.300200106* expression level of SLC16A6) + (0.177001532* expression level of HBA2) + (0.99177523* expression level of CAB39L) + (-1.037652488* expression level of DLGAP5). The median risk score was used as the basis for patient stratification. All the patients were divided into low- and high-risk groups. K-M curves were used to assess survival differences. In addition, the utility of the prognostic model was validated by the receiver operating characteristic (ROC) curve. Survival status heatmap was exhibited as expression differences in independent prognostic genes and differences in risk score distributions.

### Prognostic model validation

A nomogram with independent prognostic factors was created using the “rms” and “survival” R packages, and the accuracy of the nomogram was assessed by ROC curves, calibration curves, and decision curve analysis (DCA).

### Efficacy of chemotherapy response

The CellMiner database (https://discover.nci.nih.gov/CellMiner) was used to assess the relationship between risk score and drug sensitivity. The mRNA profiles and drug sensitivity half maximum inhibitory concentration (IC50) values of NCI-60 human cancer cell lines were obtained from the CellMiner. Subsequently, we used the CellMiner database to predict potential targeted drugs that might target the 5 BCCGs.

### Assessment of tumor immune cell infiltration

LM22 (leukocyte gene signature matrix) was obtained from the CIBERSORT database (http://CIBERSORT.stanford.edu/), which contains 547 genes that distinguish 22 human hematopoietic cell phenotypes, including seven T cell types, naïve and memory B cells, plasma cells, NK cells, and myeloid subsets. LM22 was used as a reference set to analyze tumor immune cell infiltration in BC samples, and the CIBERSORT algorithm was used to obtain the infiltration of immune cells in the low- and high-risk groups. The correlation between different tumor immune cell types and BCCGs was evaluated by Spearman’s correlation test, and the results were presented using lollipop plots.

### Evaluation of immunotherapy response

43 immune checkpoints were obtained from previous studies [37]. We compared the expression of known immune checkpoint genes in the low- and high-risk groups. The differences were statistically significant when the P value < 0.05.

### Functional enrichment analysis

The R package “ClusterProfiler” was used to identify DEGs between two risk groups and their functions were further annotated with Gene Ontology (GO) and the Kyoto Encyclopedia of Genes and Genomes (KEGG).

### Statistics analysis

To validate the independent prognostic indicators for BC, univariate and multivariate Cox regression analyses were conducted, and hazard ratios (HR) and 95% confidence intervals (CI) were reported. The prognostic performance of the risk score was evaluated using the area under the ROC curve (AUC). The Wilcoxon test analyzed the differences between the two groups’ variables. A two-sided P value of 0.05 was determined to be statistically significant. All statistical analyses were conducted using R x64 version 4.0.5.

### Data availability statement

Publicly available datasets were analyzed in this study, the data from TCGA database are available from https://portal.gdc.cancer.gov/, the data from GEO database are available from Home - GEO - NCBI (nih.gov), the data from HPA database are available from The Human Protein Atlas.
